# Echinocandin-Induced Microevolution of Candida parapsilosis Influences Virulence and Abiotic Stress Tolerance

**DOI:** 10.1128/mSphere.00547-18

**Published:** 2018-11-14

**Authors:** Csaba Papp, Katica Kocsis, Renáta Tóth, László Bodai, Jesse R. Willis, Ewa Ksiezopolska, Nancy E. Lozoya-Pérez, Csaba Vágvölgyi, Hector Mora Montes, Toni Gabaldón, Joshua D. Nosanchuk, Attila Gácser

**Affiliations:** a Department of Microbiology, Faculty of Science and Informatics, University of Szeged, Szeged, Hungary; b Department of Biochemistry and Molecular Biology, Faculty of Science and Informatics, University of Szeged, Szeged, Hungary; c Centre for Genomic Regulation (CRG), The Barcelona Institute of Science and Technology, Barcelona, Spain; d Departamento de Biología, División de Ciencias Naturales y Exactas, Campus Guanajuato, Universidad de Guanajuato, Guanajuato, Guanajuato, México; e Universitat Pompeu Fabra, Barcelona, Spain; f ICREA, Barcelona, Spain; g Division of Infectious Diseases, Department of Medicine, Albert Einstein College of Medicine, Bronx, New York, USA; h Department of Microbiology and Immunology, Albert Einstein College of Medicine, Bronx, New York, USA; i MTA-SZTE Lendület Mycobiome Research Group, University of Szeged, Szeged, Hungary; University College Dublin, Belfield

**Keywords:** *Candida parapsilosis*, echinocandins, microevolution, virulence

## Abstract

*Candida* species are a major cause of life-threatening bloodstream infections worldwide. Although Candida albicans is responsible for the vast majority of infections, the clinical relevance of other *Candida* species has also emerged over the last twenty years. This shift might be due in part to changes in clinical guidelines, as echinocandins became the first line of therapeutics for the treatment. Candida parapsilosis is an emerging non-*albicans Candida* species that exhibits lower susceptibility levels to these drugs. *Candida* species frequently display resistance to echinocandins, and the mechanism for this is well-known in C. albicans and Candida glabrata, where it is mediated by amino acid substitutions at defined locations of the β-1,3-glucan synthase, Fks1p. In C. parapsilosis isolates, Fks1p harbors an intrinsic amino acid change at position 660 of the hot spot 1 (HS1) region, which is thought to be responsible for the high MIC values. Less is known about acquired substitutions in this species. In this study, we used directed evolution experiments to generate C. parapsilosis strains with acquired resistance to caspofungin, anidulafungin, and micafungin. We showed that cross-resistance was dependent on the type of echinocandin used to generate the evolved strains. During their characterization, all mutant strains showed attenuated virulence *in vivo* and also displayed alterations in the exposure of inner cell wall components. The evolved strains harbored 251 amino acid changes, including three in the HS1, HS2, and HS3 regions of Fks1p. Altogether, our results demonstrate a direct connection between acquired antifungal resistance and virulence of C. parapsilosis.

**IMPORTANCE** Candida parapsilosis is an opportunistic fungal pathogen with the ability to cause infections in immunocompromised patients. Echinocandins are the currently recommended first line of treatment for all *Candida* species. Resistance of Candida albicans to this drug type is well characterized. C. parapsilosis strains have the lowest *in vitro* susceptibility to echinocandins; however, patients with such infections typically respond well to echinocandin therapy. There is little knowledge of acquired resistance in C. parapsilosis and its consequences on other characteristics such as virulence properties. In this study, we aimed to dissect how acquired echinocandin resistance influences the pathogenicity of C. parapsilosis and to develop explanations for why echinocandins are clinically effective in the setting of acquired resistance.

## Abstract (precis)

Candida parapsilosis is an opportunistic fungal pathogen with the ability to cause infections in immunocompromised patients. Echinocandins are the currently recommended first line of treatment for all *Candida* species. Resistance of Candida albicans to this drug type is well characterized. C. parapsilosis strains have the lowest *in vitro* susceptibility to echinocandins; however, patients with such infections typically respond well to echinocandin therapy. There is little knowledge of acquired resistance in C. parapsilosis and its consequences on other characteristics such as virulence properties. In this study, we aimed to dissect how acquired echinocandin resistance influences the pathogenicity of C. parapsilosis and to develop explanations for why echinocandins are clinically effective in the setting of acquired resistance.

## INTRODUCTION

*Candida* species are among the most common fungal pathogens isolated from hospitalized patients. The risk of candidemia is higher in intensive care units (ICUs) than in other hospital units, as the incidence of overall bloodstream *Candida* infections in ICUs is above 50%, and it is increasing relative to non-ICU units (1, 2). Candida albicans is the most common species responsible for bloodstream *Candida* infections worldwide, although the prevalence of other non-*albicans Candida* (NAC) species such as C. parapsilosis, C. glabrata, C. tropicalis, and C. krusei has markedly increased in the past two decades (1, 3–8). This phenomenon is most likely due to the widespread and increasingly common utilization of echinocandins, especially as they are currently recommended as the first-line antifungal for treatment of invasive candidiasis (9–12). After C. albicans, C. parapsilosis is the second or third most common cause of invasive candidiasis depending on the geographical location (5, 13, 14). In fact, previous exposure to echinocandins is a risk factor for C. parapsilosis infections, as this species manifests naturally higher MIC values to the three clinically available echinocandins (caspofungin [CAS], anidulafungin [AND], and micafungin [MICA]) compared to other *Candida* species (15, 16). However, patients with C. parapsilosis systemic infections respond well to echinocandin treatments, and the efficacy of echinocandin treatments is similar to that obtained with azole treatments (17, 18).

C. albicans and C. glabrata clinical isolates are usually susceptible to echinocandins, and as mentioned above, significantly lower MIC values are reported for these drugs than for C. parapsilosis. Nevertheless, an increasing number of resistant strains from each species have been reported (19, 20). In both C. albicans and C. glabrata, resistance is mainly attributable to amino acid changes in the hot spot regions (hot spot 1 [HS1] and HS2) of the β-glucan synthase Fks1 as an acquired resistance mechanism. In addition, similar changes have been reported in the hot spot regions of Fks2 in C. glabrata, which has also been associated with echinocandin resistance (21–23). Amino acid substitutions at positions Phe641, Ser645 (in HS1), and Arg1361 (in HS2) in Fks1p of C. albicans and most of the pathogenic NAC species result in echinocandin resistance. The same substitutions are also present in the Fks2p in C. glabrata (21, 22, 24). Additional alterations in the HS1 and HS2 regions of C. albicans and C. glabrata Fks1p include the occurrence of substitutions at Pro649 and Trp1358. Although these modifications result in caspofungin resistance in laboratory strains *in vitro*, they are not relevant in terms of resistance development *in vivo*, as these substitutions have not been found in clinical isolates (21, 22, 24–26). Previous studies have identified a third hot spot region (HS3) in Saccharomyces cerevisiae, where the presence of Trp695 substitution resulted in phenotypic resistance to echinocandins. However, its C. albicans equivalent, Trp697, has no clinical significance, as this alteration does not contribute to treatment failure (24, 25, 27). Such acquired mutations are the result of previous exposure to echinocandins for most of the pathogenic *Candida* species. In contrast, C. parapsilosis isolates have a naturally occurring polymorphism in Fks1, which results in reduced susceptibility to echinocandins. Specifically, Pro649 in C. albicans or the equivalent in other NAC species is substituted to Ala660 in C. parapsilosis at the region mentioned, which has been shown to contribute to higher echinocandin MIC values compared to other *Candida* species. This intrinsic substitution in this species has an unclear clinical importance, as C. parapsilosis infections generally respond well to commonly applied therapeutic echinocandin concentrations (24, 25, 28).

Previous studies revealed that C. albicans isolates with acquired Fks1 amino acid exchanges, which are also frequently associated with antifungal resistance, showed reduced fitness during *in vivo* and *in vitro* experiments. This phenomenon was explained by the reduced β-glucan synthetic activity of Fks1, which contributed to an altered cell wall structure and composition and also resulted in attenuated virulence in mice compared to an isogenic C. albicans strain (21, 22, 29). The reduced virulence due to Fks1 deficiency might be the reason for the rare horizontal transmission of echinocandin-resistant C. albicans strains between patients; however, C. parapsilosis horizontal transmission is a significant problem in ICUs, as this species is frequently isolated from the hands of health care workers (3, 30).

The mechanism and fitness cost of acquired resistance in C. albicans have been well investigated. However, our knowledge about acquired resistance mechanisms in C. parapsilosis is limited. Therefore, we set out to examine such processes in this species through the generation of *in vitro* echinocandin-evolved strains, each grown in the presence of a particular echinocandin: CAS, AND, or MICA. These directed evolution experiments were followed by virulence assays and sequence analysis of these evolved strains in order to reveal resistance mechanisms and potential alterations in their virulence.

## RESULTS

### Generation and altered susceptibility of microevolved strains.

Prior to the directed evolution experiment, MIC values for caspofungin (CAS), anidulafungin (AND), and micafungin (MICA) were determined for Candida parapsilosis CLIB 214 strain, which had MIC values of 2 µg/ml, 1 µg/ml, and 1 µg/ml, respectively (Table 1). First, we generated the adapted strains by direct selection, and then evolved strains were derived from the adapted strains by repeatedly culturing them in YPD broth to exclude resistant phenotypes due to transcriptional changes caused by the direct interaction with the different drugs. After the generation of adapted and evolved strains for each of the echinocandins, their susceptibility to azoles was also tested: including fluconazole (FLU), voriconazole (VOR), posaconazole (POS), and itraconazole (ITR). The responsiveness of each strain was further elucidated in the presence of CAS, AND, and MICA. The CAS-adapted and -evolved strains (CAS^ADP^ and CAS^EVO^, respectively) were resistant to CAS and MICA after 24 h. At 48 h, the respective strains became resistant to all three echinocandins, represented by the elevated MIC values that rose above 8 μg/ml. CAS^ADP^ and CAS^EVO^ strains further showed slightly increased MIC values to fluconazole and itraconazole. AND^ADP^ and AND^EVO^ strains were resistant to all applied echinocandins after both 24 h and 48 h. In contrast, MICA^ADP^ and MICA^EVO^ strains were resistant only to MICA and showed slightly decreased sensitivity to AND (MIC value = 4 µg/ml) compared to the parental C. parapsilosis CLIB 214 strain. The resistant phenotype was stable for all echinocandin-evolved strains (CAS^EVO^, AND^EVO^, and MICA^EVO^) at both time points, as there were no notable differences in MIC values between adapted and evolved strains.

**TABLE 1:** Distribution of MICs determined for the parental, adapted, and evolved C. parapsilosis strains

Strain	MIC (µg/ml) of antifungal:													
	FLU		VOR		POS		ITR		CAS		AND		MICA	
	24 h	48 h	24 h	48 h	24 h	48 h	24 h	48 h	24 h	48 h	24 h	48 h	24 h	48 h
CLIB 214	1	1	0.031	0.031	0.031	0.031	0.062	0.25	2	2	1	1	1	1
CAS^ADP^	2	2	0.031	0.031	0.031	0.031	0.125	0.25	>8	>8	>8	>8	>8	>8
CAS^EVO^	1	2	0.031	0.031	0.031	0.031	0.125	0.25	>8	>8	4	8	>8	>8
AND^ADP^	1	1	0.031	0.031	0.031	0.031	0.062	0.125	>8	>8	>8	>8	>8	>8
AND^EVO^	1	1	0.031	0.031	0.031	0.031	0.062	0.125	>8	>8	>8	>8	>8	>8
MICA^ADP^	1	1	0.031	0.031	0.031	0.031	0.062	0.125	2	2	4	4	>8	>8
MICA^EVO^	1	1	0.031	0.031	0.031	0.031	0.062	0.125	2	2	4	4	>8	>8

### Microevolution alters stress response in evolved strains.

During infection, pathogenic microbes have to maintain their homeostasis in order to survive in a new niche. Inside the host, a wide range of stress-inducing factors influences the viability of invading fungi. These factors include oxidative, membrane, wall, and osmotic stressors. Thus, we performed spot plate assays using YPD solid medium complemented with different stress-inducing agents.

**FIG 1:**
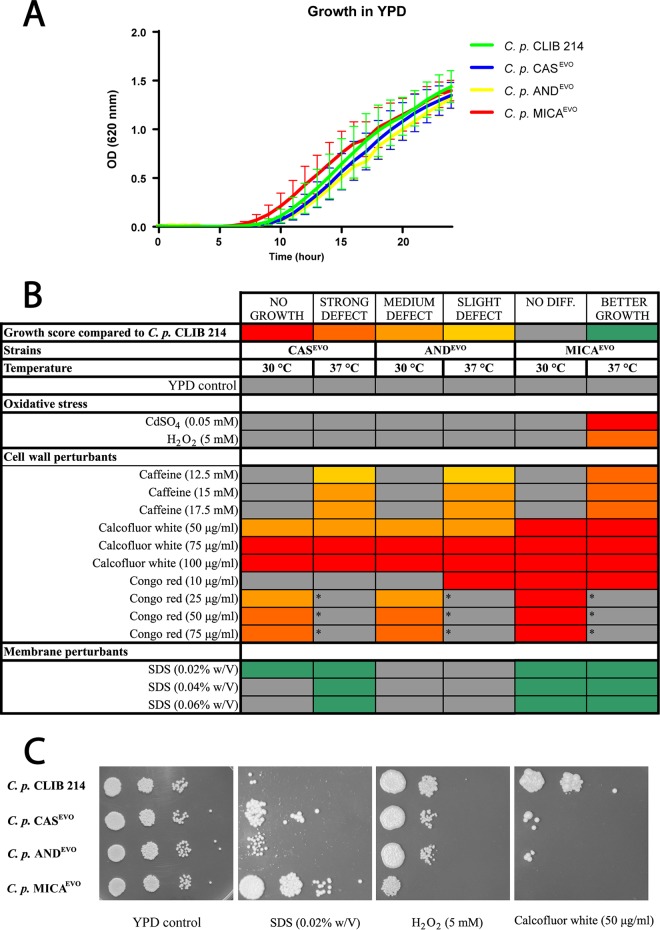
Growth of echinocandin-evolved strains in the presence of abiotic stressors. (A) Growth kinetics of parental C. parapsilosis CLIB 214 and echinocandin-evolved strains (CAS^EVO^, AND^EVO^, and MICA^EVO^). (B) Heat map of stress sensitivity and resistance to oxidative stress and cell wall and membrane perturbants. The asterisks indicate that C. parapsilosis CLIB 214 failed to grow in the presence of Congo red at 37°C, which precluded comparisons with evolved strains under this condition at this temperature. w/V, weight/volume. (C) Representative images of growth capabilities on YPD solid medium complemented with SDS, H_2_O_2_, and calcofluor white.

Generally, on YPD plates, there were no differences in the growth capabilities between the parental and evolved strains at 30°C and 37°C (Fig. 1B and C). We did not detect any restriction in growth kinetics between the parental and all echinocandin-evolved strains in YPD broth during the 24-hour incubation time in the absence of the examined stressors (Fig. 1A).

We found that the MICA^EVO^ strain was sensitive to the presence of oxidative stressors, as it was unable to grow on CdSO_4_-supplemented media and also showed a strong growth defect when H_2_O_2_ was present at 37°C (Fig. 1B and C). All evolved strains showed decreased growth capabilities on YPD plates containing cell wall-perturbing agents. At 37°C, evolved strains were able to grow, although a mild growth defect was identified in the presence of caffeine (12.5 mM, 15 mM, and 17.5 mM), indicating temperature-dependent alterations in the TOR signaling pathway (31). No such defect was observed at 30°C (Fig. 1A). In the presence of calcofluor white, the strains were unable to grow or showed a severe growth defect at both 30°C and 37°C (Fig. 1B and C). Similar defects were detected in all evolved strains on plates supplemented with ≥25 μg/ml Congo red at 30°C. Notably, at these concentrations, the parental C. parapsilosis CLIB 214 strain was also unable to grow at 37°C (Fig. 1B, indicated by asterisks). In contrast with the oxidative agents and cell wall perturbants, CAS^EVO^ and MICA^EVO^ strains showed increased fitness in the presence of the membrane-damaging compound sodium dodecyl sulfate (SDS) compared to the CLIB 214 strain (Fig. 1B and C).

### Acquired resistance to echinocandins resulted in attenuated virulence *in vivo*.

**FIG 2:**
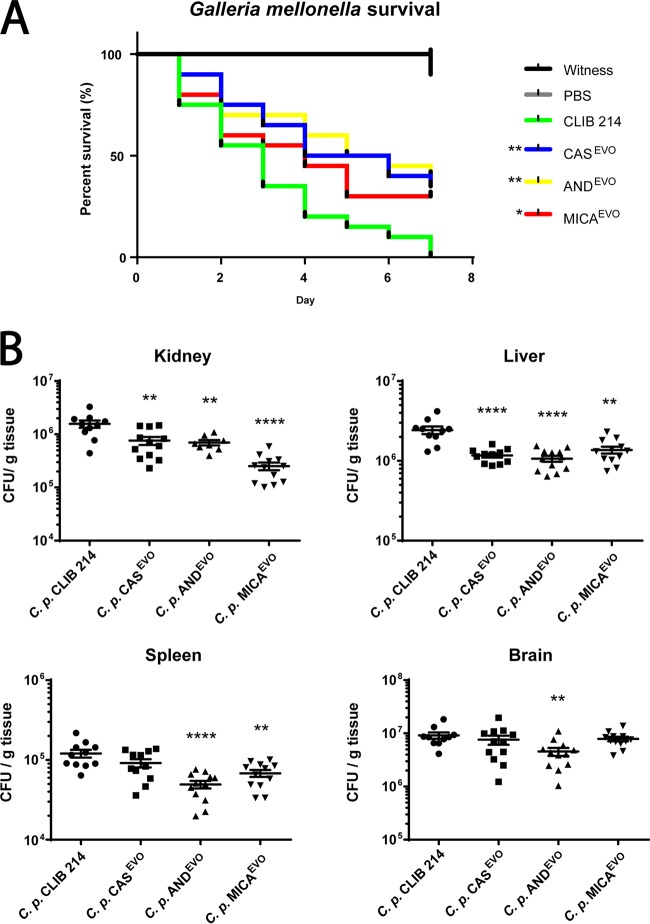
*In vivo* virulence properties of C. parapsilosis CLIB 214, CAS^EVO^, AND^EVO^, and MICA^EVO^ strains. (A) Survival proportion of *Galleria mellonella* infected with the parental strain and echinocandin-evolved strains. Experiments were repeated two times, and 20 larvae/group/experiment were used. Significant differences were determined with log rank tests. Two controls were run: PBS-treated (uninfected) and witness control (no injection, uninfected). (B) Fungal burden in the organs of BALB/c mice infected with CLIB 214, CAS^EVO^, AND^EVO^, or MICA^EVO^ strain, represented in CFU/gram of tissue 3 days postinfection. Data points are means ± SEM pooled from three independent experiments (four BALB/c mice/group/experiment). Significant differences were determined by Mann-Whitney U-tests. *, *P* ≤ 0.05; **, *P* ≤ 0.005; ****, *P* ≤ 0.0001.

To investigate potential changes in the virulence properties of the CAS^EVO^, AND^EVO^, and MICA^EVO^ strains, we utilized Galleria mellonella (as a nonmammalian, alternative model) to study disseminated candidiasis. The susceptibility of wax moth larvae to C. parapsilosis CLIB 214, CAS^EVO^, AND^EVO^, or MICA^EVO^ strains was examined by determining larval survival rates. As a result, we found that the survival rates of CAS^EVO^-, AND^EVO^-, or MICA^EVO^-infected larvae were higher than those inoculated with CLIB 214 cells (Fig. 2A). No deaths occurred in the uninfected or PBS-injected larvae during the study period.

To confirm our findings on the attenuated virulence of echinocandin-evolved strains, we also determined fungal burdens in the kidneys, livers, spleens, and brains of BALB/c mice 3 days after infection with the evolved strains and the reference C. parapsilosis CLIB 214 strain. The fungal burdens in the kidneys and livers of mice infected with echinocandin-evolved strains were lower than those of CLIB 214-infected mice. Fungal CFU recovered from the spleen were significantly lower in the case of the AND^EVO^- and MICA^EVO^-infected mice than for CAS^EVO^-infected mice. CFU retrieved from CAS^EVO^-challenged mice were similar to those obtained with the reference strain. AND^EVO^-infected mice had the lowest fungal burden in the brain compared to all other C. parapsilosis strains (Fig. 2B).

All of these results suggest that the CAS^EVO^, AND^EVO^, and MICA^EVO^ strains are less virulent *in vivo* in both applied animal models.

### Echinocandin microevolution affects the exposure, but not the ratio, of inner cell wall components.

**FIG 3:**
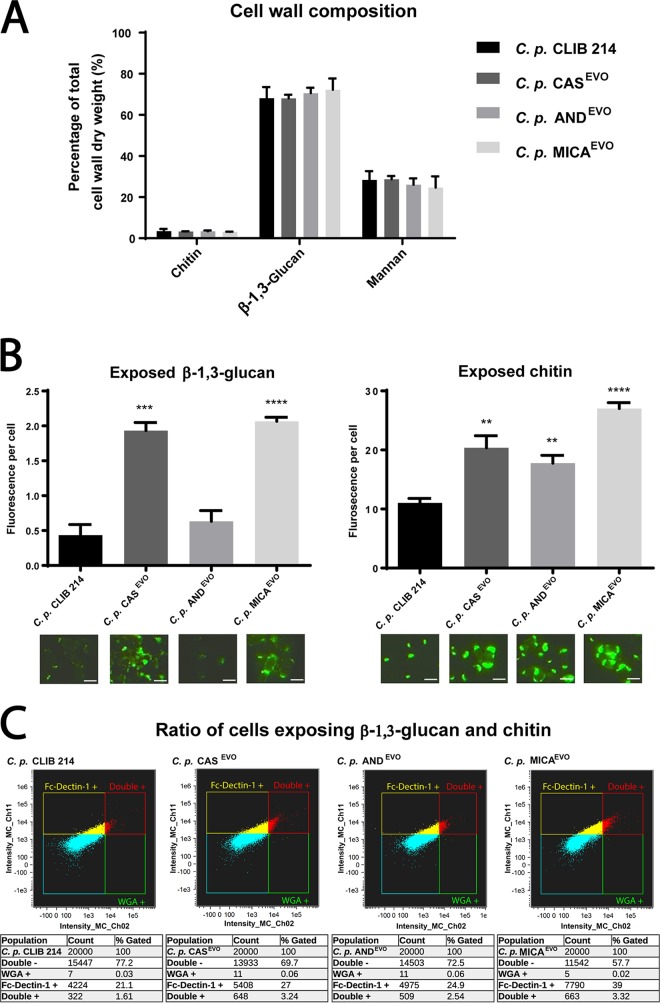
Altered cell wall structure of C. parapsilosis echinocandin-evolved strains. (A) Relative amount of chitin (*N*-acetylglucosamine), glucan, and mannose to the total cell wall weight (dry weight). Statistical analysis was performed using two-way ANOVA. (B) Semiquantitative analysis of exposed β-glucans and chitin levels. Exposure is expressed in mean fluorescence intensity/fungal cell. Microscopic images represent examples of β-1,3-glucan and chitin exposure (bars, 5 µm). Significance is determined by unpaired *t* test. (C) Flow cytometric analysis of proportions of inner cell wall component-exposing cells of CLIB 214, CAS^EVO^, AND^EVO^, and MICA^EVO^ strains. A total of 2** × **10^4^ cells were analyzed for each strain. WGA+ cells exposing chitin and Fc-Dectin-1+ cells exposing β-glucan were studied. Gates for data analysis were defined by excluding the unstained control cells of each strain. **, *P* ≤ 0.005; ***, *P* ≤ 0.0005; ****, *P* ≤ 0.0001.

To determine the cell wall composition of the C. parapsilosis parental and evolved strains, their cell walls were purified and acid hydrolyzed, followed by an analysis using high-performance anion-exchange chromatography with pulsed amperometric detection (HPAEC-PAD). Our results showed that there were no changes in the ratio of cell wall components in the echinocandin-evolved strains compared to the parental strain (Fig. 3A).

To evaluate the exposure of chitin and β-1,3-glucan, cells of the parental and evolved strains were stained with fluorescently labeled Fc-Dectin-1 (binds β-1,3-glucan) and WGA (binds chitin), and the mean fluorescence intensity was determined by microscopy. We found that the exposure of chitin and β-1,3-glucan—present in the inner cell wall layer—was markedly altered in the echinocandin-evolved strains compared to the reference strain. In the CAS^EVO^ and MICA^EVO^ strains, chitin and β-1,3-glucans were significantly more exposed than in the parental strain. Interestingly, in the AND^EVO^ strain, chitin was also exposed similarly to the other echinocandin-evolved strains; however, β-1,3-glucan exposure was similar to that of the reference strain (Fig. 3B).

We also defined the ratio of cells exposing chitin; β-1,3-glucan or both using WGA-FITC and Fc-Dectin-1/Alexa Fluor 647-labeled anti-human IgG1Fc staining. The proportions of β-1,3-glucan-exposing cells were 22.71%, 30.24%, 27.44%, and 42.32%, while the rates of chitin-exposing cells were 1.64%, 3.3%, 2.6%, and 3.34% in CLIB 214, CAS^EVO^, AND^EVO^, and MICA^EVO^ strains, respectively. These data suggest that the number of cells exposing inner cell wall layers was significantly higher in the CAS^EVO^ and MICA^EVO^ strains than in the parental strain. Although chitin exposure of AND^EVO^ cells was higher than in CLIB 214 cells, β-1,3-glucan exposure of these cells was similar to that observed in the parental strain (Fig. 3C).

### Echinocandin microevolution does not affect phagocytosis or phagolysosome colocalization.

**FIG 4:**
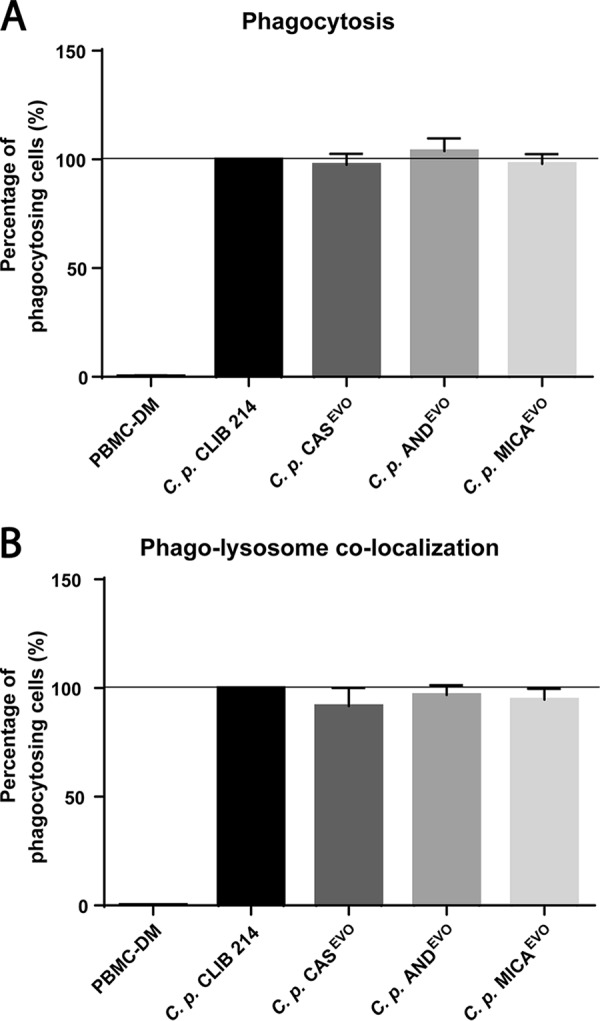
Flow cytometric analysis of phagocytosis and phagosome maturation in PBMC-DMs after infection with C. parapsilosis CLIB 214, CAS^EVO^, AND^EVO^, or MICA^EVO^ strain. (A) Phagocytosing PBMC-DMs normalized to the phagocytic activity were measured and compared to the parental CLIB 214 strain. (B) Ratio of cells with phagolysosome colocalization, normalized to the proportion of phagosome maturation after the uptake of CLIB 214 cells. For each experiment, PBMC-DMs were utilized from five healthy human donors. A total of 2** × **10^4^ PBMC-DMs were analyzed per donor for each strain. Significant differences were determined using Wilcoxon signed rank tests.

To determine the ratio of actively phagocytosing human peripheral blood mononuclear cell-derived macrophages (PBMC-DMs) s and the extent of phagolysosome colocalization after 2 h of incubation, C. parapsilosis CLIB 214 and echinocandin-evolved strains were stained with Alexa Fluor 647 (phagocytosis) and pHrodo red (phagolysosome fusion). Stained strains were then coincubated with PBMC-DMs. Our results revealed no significant differences in terms of phagocytosis or in phagosome maturation between the parental CLIB 214 strain and echinocandin-evolved strains (Fig. 4A and B).

### Microevolution in the presence of echinocandins is possibly due to acquired amino acid substitutions in C. parapsilosis Fks1.

**FIG 5:**
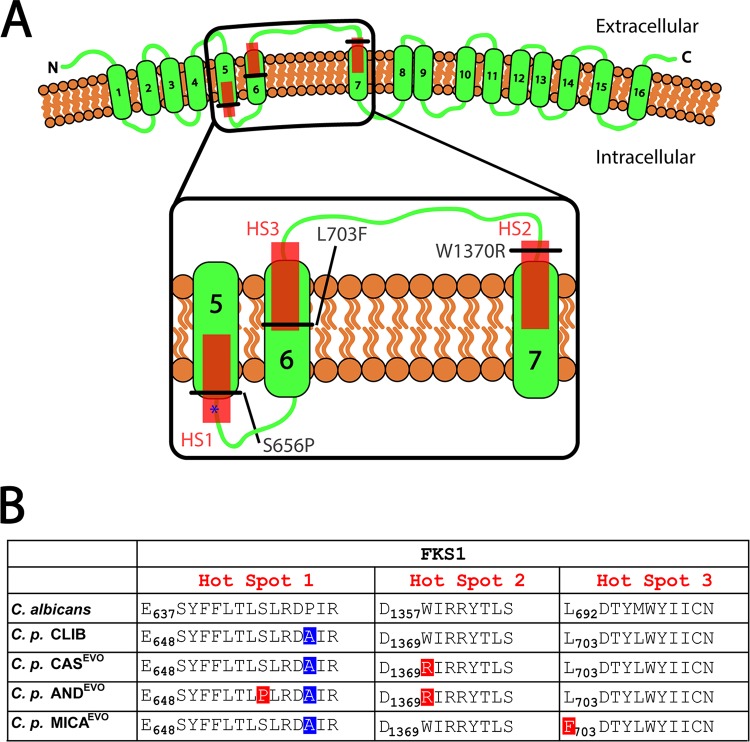
Predicted topology of C. parapsilosis Fks1p and summary of all amino acid alterations found in the three echinocandin-evolved strains. (A) Schematic topology of the Fks1 protein in the plasma membrane. The N terminus (N) and C terminus (C) of Fks1p are indicated. Transmembrane segments of Fks1 protein (green rectangles) and hot spot regions of Fks1 protein (red squares) are shown. The black lines indicate the positions of all amino acid substitutions in all three examined echinocandin-evolved strains. (B) Aligned protein sequences of HS1, HS2, and HS3 regions in C. albicans, C. parapsilosis CLIB 214, CAS^EVO^, AND^EVO^, and MICA^EVO^ strains. Acquired amino acid substitutions in the echinocandin-evolved strains are shown on red squares. Intrinsic amino acid changes naturally present in all C. parapsilosis clinical isolates are shown on blue squares.

Whole-genome comparison was performed on the genomic DNA of the parental and echinocandin-evolved strains. SNPs identified in the whole-genome sequence analysis are listed in Table S1 in the supplemental material. During whole-genome analyses, we identified point mutations in the CPAR2_106400 gene of echinocandin-evolved strains at the contig positions 1374083, 1376082, and 1376225, where the following amino acid substitutions occurred: W1370R (tryptophan to arginine), L703F (leucine to phenylalanine), and S656P (serine to proline), respectively (Fig. 5A). The analyzed genomes presented other nonsynonymous mutations (134, 96, and 153, total number of nonsynonymous mutations in CAS_S3, AND_S2, and MIC_S1, respectively), CPAR2_106400 was one of 38 genes that accumulated nonsynonymous mutations in parallel in the three evolved strains and harbored an average of 1.2% of the nonsynonymous mutations observed in the three experiments. Only seven other genes had a higher relative number of nonsynonymous mutations, these genes coded for two proteins of unknown function (CPAR2_101640, CPAR2_402490, 1.4%, 1.7%), one putative transporter (CPAR2_301640, 1.4%), and three putative cell membrane or cell wall proteins (CPAR2_600430, CPAR2_300110, and CPAR2_806400, CPAR2_303790, with 1.8%, 1.6%, 1.2%, and 1.3%, respectively). Although mutations in those other genes may also be related to adaptation to the exerted selective pressure, given the known involvement of CPAR2_106400 in echinocandin resistance, we focused further on this gene. CPAR2_106400 is an orthologous gene of C. albicans *FKS1* gene (alias *GSC1*) encoding the main component of the β-1,3-glucan synthase complex. There was no nucleic acid sequence variation in any of the echinocandin-evolved strains in the CPAR2_109680 and CPAR2_804030 genes, which are orthologues of C. albicans *GSL1* and *GSL2* and bearing β-1,3-glucan synthase activity. The identified substitutions occurred at different positions of Fks1 in the echinocandin-evolved strains. The W1370R amino acid substitution occurred at the HS2 region in the CAS^EVO^ and AND^EVO^ strains in a homozygous form. Further, the AND^EVO^ strain was also found to harbor a S656P substitution in a heterozygous form at the HS1 region. In the MICA^EVO^ strain, only L703F was identified at HS3 as a homozygous substitution (Fig. 5B).

10.1128/mSphere.00547-18.2 TABLE S1 SNPs identified in the whole-genome sequence analysis. Only mutations that differ between the parental strain and at least one of the evolved strains is shown, as these are the mutations that appeared during our experiment. Columns in the table indicate, in this order: Coordinates, contig number and position within contig of the given SNP; Ref, allele in the reference strain; Genomic region, exonic or intergenic; Gene, gene name when applicable; AA_change, when applicable, of the format XYZ, where X is the reference amino acid, Y is the position within the translated protein sequence, and Z is the alternative amino acid; [sample_name], when a SNP is present, of the format a;b;c;d, where a is the alternate allele, b is the zygosity, c is the coverage at the position, and d is SNP class (when applicable); Function, annotated gene function, when applicable. The first datasheet contains all original exoinic and intergenic variants mapped to the reference genome in CAS^EVO^, AND^EVO^, MIC^EVO^, and CLIB 214. The second datasheet contains all variants in the three evolved strains compared to CLIB 214. The third, fourth, and fifth datasheets contain the same as the second datasheet but individually for the specific evolved strain (CAS^EVO^, AND^EVO^, and MIC^EVO^, respectively). Download Table S1, XLS file, 0.4 MB. Copyright © 2018 Papp et al. 2018 Papp et al. This content is distributed under the terms of the Creative Commons Attribution 4.0 International license.

The topology of Fks1p in the plasma membrane of C. parapsilosis is shown in Fig. 5A. To assess the relative locations of conserved hot spot (HS) regions and defined amino acid substitutions, we used TMHMM and PRO-TMHMM as online bioinformatic tools. Using the given algorithms, we predicted 16 transmembrane helices with extracellular N and C termini (Fig. 5A). According to the prediction, HS2 and HS3 are localized at the extracellular region of the 7th and 6th transmembrane helices, respectively. The HS1 region is located at the intracellular half of the 5th transmembrane helix. The S656P substitution present in the AND^EVO^ strain appeared inside the HS1 region of Fks1. Notably, this exchange occurred close to the intrinsic P660A substitution that is a characteristic of C. parapsilosis (Fig. 5A, blue star). In the Fks1p of the MICA^EVO^ strain, the L703F amino acid exchange occurred at the N-terminal region of HS3. Further, the W1370R substitution, identified in both AND^EVO^ and CAS^EVO^ strains, was found to be located in the extracellular part of HS2, outside the 7th TM helix (Fig. 5A).

## DISCUSSION

In this study, we aimed to generate three independent echinocandin-evolved C. parapsilosis strains, each adapted to the presence of one of the three most clinically used echinocandins. Using a series of growth steps in complex media supplemented with CAS, AND, and MICA in stepwise elevated concentrations, we successfully generated C. parapsilosis CAS^EVO^, C. parapsilosis AND^EVO^, and C. parapsilosis MICA^EVO^ strains with acquired and stable resistance to the corresponding echinocandins. Previous studies have shown that C. parapsilosis is able to acquire resistance to echinocandins and exposure to these antifungals also influences azole susceptibility (32). Interestingly, in this study, we were not able to show cross-resistance between echinocandins and azoles upon examination of the generated echinocandin-microevolved strains. This could be explained by the restricted number of amino acid substitutions in the evolved strains. Only the CAS^EVO^ strain showed slightly elevated MIC values to fluconazole and itraconazole; however, it was still considered susceptible to the azole antifungals, as the obtained values did not reach the cutoff values for resistance set by the Clinical and Laboratory Standards Institute (CLSI). However, similar to other studies (33), we were able to detect cross-resistance between echinocandins, specifically in the case of CAS^EVO^ and AND^EVO^ strains. These strains were resistant to all three echinocandins. However, the CAS^EVO^ strain showed an increased MIC value to AND, but according to Rosenberg et al., this elevated MIC value of CAS^EVO^ strain is most probably tolerant to AND rather than resistant (34). The MICA^EVO^ strain showed resistance to MICA only.

During the characterization of the microevolved strain, MICA^EVO^ showed the most divergent phenotype in response to abiotic stress, being the most sensitive to oxidative stress and cell wall-perturbing agents and resistant to SDS-driven membrane damage. Notably, the CAS^EVO^ and AND^EVO^ strains were also shown to be sensitive to the presence of cell wall stressors.

Previously, Ben-Ami et al. (29) demonstrated that acquired resistance to echinocandins results in fitness costs that ultimately lead to attenuated virulence in C. albicans clinical isolates. Similarly, in this study, we showed that echinocandin-evolved strains also displayed decreased virulence *in vivo*, as represented by higher survival rates of G. mellonella larvae and the low number of CFU recovered from mice after challenge with the generated strains compared to the parental strain.

In clinically relevant *Candida* species, increased chitin content in the cell wall is a short-term adaptation strategy of fungal cells upon exposure to echinocandins prior to *FKS1-2*-mediated resistance mechanisms (25). Alterations in the chitin content of certain strains of different *Candida* species also contribute to increased MIC values to echinocandins (35). Interestingly, in the echinocandin-evolved strains, we did not observe any significant changes in the amounts of major cell wall components compared to those of the reference strains. However, the inner cell wall layers were significantly more exposed on the cell surfaces of microevolved strains, and the ratio of chitin and β-1,3-glucan-exposing cells was also higher than in the parental strain. Notably, β-1,3-glucan exposure was not distinguishable between the AND^EVO^ and the reference strain, although the strain’s attenuated virulence remained similar to, if not more prominent, than those of the other evolved strains *in vivo*.

A previous study showed that the lack of mannose in the outermost layer of the fungal cell wall increases the velocity of phagocytosis of C. albicans cells (36). According to a recent study, exposure of β-1,3-glucan on the surfaces of C. parapsilosis cells does not affect the phagocytic activity of human PBMC-DMs *in vitro*; however, it does *in vivo.* In their study, Perez-Garcia et al. showed that *och1Δ* cells, exposing larger amounts of β-1,3-glucan on their surfaces, are eliminated more efficiently than wild-type cells in a mouse model of invasive candidiasis (37). Our results are in accordance with both the *in vitro* and *in vivo* findings of Perez-Garcia et al., as altered chitin/β-1,3-glucan exposure of the microevolved strains does not affect the strains’ virulence *in vitro*, which is confirmed by the phagocytosis and phagolysosome maturation results, although attenuated virulence occurs *in vivo*, proven by decreased mortality rates in G. mellonella larvae, as well as the lower CFU recovered from mice after injection.

In other *Candida* species and S. cerevisiae, hot spot regions of Fks1 are highly conserved; however, in C. parapsilosis, a species-specific intrinsic substitution is present at amino acid position 660, which is occupied by alanine in C. parapsilosis (25). In certain C. parapsilosis clinical isolates, additional substitutions have recently been reported in Fks1 (V595I and F1386S), but these changes are localized outside the well-known hot spot regions (38). The echinocandin-evolved strains, generated in this study, revealed additional, yet unidentified amino acid substitutions in the HS1, HS2, and HS3 regions of the corresponding protein in this species. A W1370R (tryptophan-to-arginine) amino acid exchange was detected in the CAS^EVO^ and AND^EVO^ strains that might be equivalent to substitutions at Trp1358 in C. albicans and C. glabrata, although in these species, the amino acid exchanges cause only weak resistance. In this study, in 2 out of the 3 C. parapsilosis strains, the corresponding substitution may be responsible for a strong cross-resistance to echinocandins (21, 22). Although similar mutations in other *Candida* species suggest that this is the cause of resistance, confirmation with directed mutation experiments is still required to confirm this in C. parapsilosis. In the AND^EVO^ strain, the S656P substitution was present only in a heterozygous form; however, it appeared relevant in AND resistance, as the MIC value of this strain increased markedly compared to the MIC of the CAS^EVO^ strain, despite the fact that both of these strains harbor a W1370R substitution. In C. albicans, the same amino acid exchange results in a strong echinocandin-resistant phenotype *in vitro*, which has also been reported to result in attenuated virulence of the corresponding clinical isolates (29). The MICA^EVO^ strain harbored a L703F substitution within the HS3 consensus region, which is another region in the Fks1 protein (27). This substitution is a relatively novel finding, as in C. albicans, only Trp697 has been identified as an exchange event in the HS3 region (27).

We further predicted the topology of C. parapsilosis Fks1p in the plasma membrane using *in silico* tools and found that the loop connecting the 5th and 6th TM segments is probably intracellular and the loop connecting the 6th and 7th TM segments is possibly extracellular in this protein. Interestingly, in S. cerevisiae Fks1p, these loops are localized in the opposite direction (27). Our findings indicate that the resistance pattern of the echinocandin-evolved strains supports the predicted topology of C. parapsilosis Fks1p: W1370R caused the most abundant echinocandin cross-resistant phenotype, and it is located in the extracellular part of the 6th and 7th TM segment connecting loop. Additionally, the L703F substitution resulted in resistance only to MICA but not to AND and CAS. Even so, further investigations are required to generate the exact topology and structure of C. parapsilosis Fks1p.

Taken together, in this study, we have revealed a direct connection between acquired resistance and attenuated virulence in C. parapsilosis. These data provide exciting information that supports the pursuit of further studies aiming to explore and further exploit the relevance of amino acid substitutions in β-1,3-glucan synthase proteins during echinocandin resistance development and their effect on virulence regulation in other clinically relevant *Candida* species.

## MATERIALS AND METHODS

### Generation of C. parapsilosis echinocandin-evolved strains.

We generated echinocandin-evolved strains as described previously (39), with minor modifications. First, we determined the MIC values of caspofungin (CAS), anidulafungin (AND), and micafungin (MICA) for the C. parapsilosis CLIB 214 strain (40). We used CLIB 214 as the parental strain for both microevolution and later characterization studies. Three individual cultures of the CLIB 214 strain were adjusted to a final absorbance of 0.1 (λ = 640 nm) in 10-ml Sabouraud glucose broth (SGB) (4% glucose, 1% peptone). For microevolution, we aimed to apply echinocandin concentrations according to the determined MIC values. In each case, half of the MIC determining concentrations were applied at the first step of adaptation.

After incubation at 30°C for 10 h without any supplements, cells were incubated for an additional 14 h in the presence of CAS, AND, or MICA (1-µg/ml, 0.5-µg/ml, or 0.5-µg/ml concentrations, respectively). Henceforth, cells were cultured three times in fresh SGB medium containing the appropriate drugs at 1-µg/ml, 0.5-µg/ml, and 0.5-µg/ml concentrations for 24 h in the above-mentioned concentrations. After the last 24 h of incubation, absorbance of the cultures was adjusted to 0.1 in 10 ml SGB containing the appropriate echinocandin in the same concentrations mentioned above and incubated for 10 h at 30°C with the appropriate drugs (with the same concentrations). Then, CAS, AND, or MICA was added to the appropriate culture with elevated concentrations (2-fold increase for each), and cultures were grown for an additional 14 h. After that, cultures were collected and inoculated three times into fresh SGB medium containing the mentioned increased amounts of drugs and incubated for an additional 24 h. The concentration of each echinocandin was doubled on every fourth day, until reaching the final concentration of 16 µg/ml. Aliquots containing 50 µl of each culture were plated to yeast peptone dextrose (YPD) (0.5% yeast extract, 1% peptone, 1% glucose) plates complemented with 16 µg/ml caspofungin, anidulafungin, or micafungin, respectively. As a result, we obtained C. parapsilosis CAS-adapted (CAS^ADP^), AND-adapted (AND^ADP^) and MICA-adapted (MICA^ADP^) strains. Single colonies of CAS^ADP^, AND^ADP^, and MICA^ADP^ strains were subcultured 10 times in SGB without echinocandins each time for 24 h. Henceforth, these strains were referred to as echinocandin-evolved strains (CAS^EVO^, AND^EVO^, and MICA^EVO^, respectively). Figure S1 in the supplemental material summarizes the directed evolutionary process.

10.1128/mSphere.00547-18.1 FIG S1 Workflow of C. parapsilosis echinocandin-adapted and -evolved strain generation. Download FIG S1, TIF file, 1.1 MB. Copyright © 2018 Papp et al. 2018 Papp et al. This content is distributed under the terms of the Creative Commons Attribution 4.0 International license.

### Strains and culture conditions.

All strains used in this study are listed in Table 2. All C. parapsilosis strains were cultured in YPD broth, and on the next day, 200-µl aliquots were inoculated into fresh YPD medium before every experiment. C. parapsilosis CLIB 214 was maintained on YPD agar plates (supplemented with 1.5% agar), and the CAS^EVO^, AND^EVO^, and MICA^EVO^ strains were maintained on YPD solid medium supplemented with 16 µg/ml CAS, AND, and MICA, respectively.

**TABLE 2:** C. parapsilosis strains used in this study

Strain	Origin	Reference
CLIB 214	Laboratory type strain	40
CAS^ADP^	CLIB 214	This work
AND^ADP^	CLIB 214	This work
MICA^ADP^	CLIB 214	This work
CAS^EVO^	CAS^ADP^	This work
AND^EVO^	AND^ADP^	This work
MICA^EVO^	MICA^ADP^	This work

### Antifungal susceptibility testing.

MIC values were determined according to the M27-A3 protocol (41), and interpretation of MIC values was defined by the M27-S4 supplementary document (42). MICs were measured in RPMI 1640 with MOPS with L-Gln and without NaHCO_3_ (catalog no. 04-525F; Lonza) after 24 h and 48 h. MIC values for echinocandins were defined as the lowest concentrations that resulted in at least 50% growth reduction. Azoles (fluconazole [FLU], voriconazole [VOR], posaconazole [POS], and itraconazole [ITR]; Sigma-Aldrich) and echinocandins (CAS from Sigma-Aldrich; AND and MICA from MedChem Express) were used to test the susceptibility of C. parapsilosis strains.

### Determination of abiotic stressor tolerance by spot assay and growth capabilities under no-stress condition.

Synchronized suspensions of C. parapsilosis CLIB 214, CAS^EVO^, AND^EVO^, and MIC^EVO^ strains were serially diluted, and 10^4^, 10^3^, 10^2^, and 10^1^ cells were transferred to YPD solid plates adjusted to pH 4, pH 5, pH 6, pH 7, or pH 8 using McIlvine buffer and to YPD plates without any supplements as a control. For comparing growth capabilities of the four strains in the presence of osmotic and oxidative stressors as well as cell membrane- and cell wall-perturbing agents, we prepared YPD agar plates complemented with 8% (wt/vol), 10% (wt/vol), 12% (wt/vol) glycerol; 1 M and 1.5 M NaCl; 1 M and 1.5 M sorbitol (as osmotic stressors); 0.05 mM CdSO_4_; 5 mM and 10 mM H_2_O_2_ (as oxidative stressors); 12.5 mM, 15 mM, and 17.5 mM caffeine; 50 µg/ml, 75 µg/ml, and 100 µg/ml calcofluor white, 10 µg/ml, 25 µg/ml, 50 µg/ml, and 75 µg/ml Congo red (as cell wall-perturbing agents); 0.02% (wt/vol), 0.04% (wt/vol), and 0.06% (wt/vol) sodium dodecyl sulfate (SDS) (as a membrane-perturbing agent). The plates were incubated at 30°C and 37°C for 48 h. The growth scores of the evolved (EVO) strains were determined compared to the parental C. parapsilosis CLIB 214 strain. All experiments were repeated two times. We defined the defect scores as follows: a score of 1 for a strong defect such as reduced growth (smaller colonies or lower colony numbers) in the given evolved strain spot, which was three times more concentrated than the most diluted CLIB 214 spot where growth appeared; a score of 2 for a medium defect (when similar CFU appeared in the given evolved strain spot, which was two times more concentrated then the most diluted CLIB 214 spot where growth appeared); a score of 3 for a slight defect (reduced growth in the given evolved strain spot at one time the concentration of the most diluted CLIB 214 spot where growth appeared, or the presence of smaller colonies compared to the parental strain’s colonies).

We inoculated 200 µl YPD in 96-well plates with 2 × 10^3^ cells of each strain and monitored the optical density (OD) of wells for 24 h to determine the growth kinetic without stressors.

### Isolation of peripheral blood mononuclear cells (PBMCs) and differentiation of PBMC-derived macrophages (PBMC-DMs).

Human PBMCs were isolated from buffy coat (Hungarian National Blood Transfusion Service) from healthy donors by Ficoll Paque Plus (GE Healthcare) gradient centrifugation as described previously (43). PBMCs were washed with ice**-**cold phosphate-buffered saline (PBS) **(**137 mM NaCl, 2.7 mM KCl, 10 mM Na_2_HPO_4_, 2 mM KH_2_PO_4_ **[**pH 7.4]). Isolated PBMCs were suspended in RPMI 1640 supplemented with 1% 100× penicillin-streptomycin solution (Pen-Strep; Sigma-Aldrich) and the concentration of cells had been adjusted to 2 × 10^7^/ml; 2 × 10^7^, 10^7^, or 5 × 10^5^ PBMCs were transferred to 12-, 24-, or 96**-**well plates, respectively. After 1.5 h of incubation (5% CO_2_, 37°C, 100% relative humidity), floating cells were removed by changing the culture medium to AIM-V cell culturing medium (Gibco) supplemented with 10 ng/ml granulocyte-macrophage colony-stimulating factor (GM-CSF) **(**Sigma-Aldrich) every other day for 7 days.

### Phagocytosis and phagolysosome colocalization (flow cytometry).

In order to analyze the phagocytic activity and phagolysosome colocalization of PBMC-DM cells by flow cytometry, fungal cells were labeled with Alexa Fluor 647 succinimidyl ester and pHrodo red succinimidyl ester (Invitrogen) as follows. First, 22 µl Na_2_CO_3_ (1 M, pH 10), 4 µl Alexa Fluor 647 (1 mg/ml in DMSO), and 4 µl pHrodo red (100 µg/ml in DMSO) were added to 200-µl fungal cell suspensions in Hanks’ balanced salt solution (HBSS) (Lonza) and incubated for 1 h in the dark at room temperature. pHrodo red stains the fungal cell wall and emits fluorescent light only in a highly acidic environment such as the phagolysosome. Fungal cells were then washed four times with HBSS, and cell concentrations were adjusted to the appropriate concentration. PBMC-DM cells were infected with the labeled fungal cells at a 1:5 ratio in 12-well cell culture plates and incubated for 2 h (5% CO_2_, 37°C, 100% relative humidity). After the incubation, extracellular fungal cells were removed by washing the wells with PBS. Macrophages were harvested from the wells with trypsin (5 mg/ml; Sigma-Aldrich). Samples were measured in PBS with a FlowSight instrument (Amnis), and data were analyzed with the IDEAS 6.2 software.

### *In vivo* infection of mice and fungal burden.

For determination of fungal burden, 8- to 12-week-old female BALB/c (BRC, Szeged, Hungary, XVI./2015) mice were infected via the lateral tail vein with 2** × **10^7^ yeast cells in 100 µl PBS (*N* ≥ 11 per C. parapsilosis strain). Three days postinfection, animals were euthanized, and the livers, spleens, kidneys, and brains were collected surgically, weighed, and homogenized in an Ultra-Turrax T25 homogenizer (Sigma). Organ homogenates were plated to YPD agar supplemented with 1% Pen-Strep, and the numbers of colony-forming units (CFU) were determined after 48-h incubation at 30°C.

### Ethic statement.

Animal experiments were performed according to the Hungarian national (1998. XXVIII; 40/2013) and European (2010/63/EU) animal ethic guidelines. Procedures were licensed by the Animal Experimentation and Ethics Committee of the Biological Research Centre of the Hungarian Academy of Sciences and the Hungarian National Animal Experimentation and Ethics Board (clearance number XVI./03521/2011) with the University of Szeged granting permission XII./00455/2011 and XVI./3652/2016 to work with mice.

For isolation of PBMCs, blood samples were taken from healthy donors. This procedure and the respective consent documents were approved by the Institutional Human Medical Biological Research Ethics Committee of the University of Szeged. All healthy donors provided written informed consent. All experiments were performed in accordance with the guidelines and regulations of the Ethics Committee of the University of Szeged, and experimental protocols were approved by this institutional committee.

### Survival of Galleria mellonella larvae.

*G. mellonella* larvae were inoculated with 5** × **10^7^ yeast cells in 10 µl PBS via the last proleg using a Hamilton syringe with a cone-tipped 26-gauge needle (Sigma-Aldrich). For each C. parapsilosis strain, 20 wax moth larvae were infected. For PBS-treated (uninfected) and witness control (no injections, uninfected), 15 animals were utilized. Larvae were maintained at 30°C, and the survival of larvae was monitored daily.

### Analysis of cell wall composition.

Cells were mechanically broken in a fastprep homogenizer, cell debris were removed by centrifuging at 20,000 × *g* for 5 min at 4°C, and the walls were collected and cleared by centrifuging at least six times with deionized water. Then, the walls were cleaned by serial incubations with hot SDS, β-mercaptoethanol, and NaCl and hydrolyzed with hot 2 M trifluoroacetic acid as described previously (44). Acid-hydrolyzed samples were analyzed by high-performance anion-exchange chromatography with pulsed amperometric detection (HPAEC-PAD) in a Dionex system (Thermo Fisher Scientific) as described earlier (45).

### Fluorochrome staining for chitin and β-glucan exposure (microscopy, flow cytometry).

Chitin labeling was performed by incubating cells with 1 mg/ml WGA-FITC (Sigma) for 1 h at room temperature, as reported earlier (46). For fluorescent detection of β-1,3-glucan, cells were first incubated with 5 µg/ml IgG Fc-Dectin-1 chimera (47) for 40 min at room temperature and then further incubated with 1 µg/ml donkey anti-Fc IgG-FITC (Sigma) for 40 min at room temperature (48). Cells were examined by fluorescence microscopy using a Zeiss Axioscope-40 microscope and an Axiocam MRc camera. From the pictures acquired, the fluorescence associated with 300 cells was calculated using the software program Adobe Photoshop CS6 and the following formula: [(total of green pixels − background green pixels) × 100]/total pixels.

### Genome sequencing.

Genomic DNA sequencing libraries were prepared using Nextera XT DNA Library Preparation kit (Illumina) with Nextera XT Index kit adapters following the manufacturer’s recommendations for sequencing runs ≥ 2 × 250 cycles. Sequencing libraries were validated and quantified using an Agilent 2100 Bioanalyzer capillary electrophoresis instrument with Agilent DNA 1000 kit. Whole-genome sequencing was performed with an Illumina MiSeq sequencer using MiSeq reagent kit v3 (600 cycles) according to the manufacturer’s instructions. The read length was 2 × 300 bp, and the final per base sequencing depth ranged from 46 to 130× (CLIB_S4: 51; AND_S2: 56; MIC_S1: 130; CAS_S3:46).

### Genome analysis of C. parapsilosis strains.

Paired-end fastq reads files were first trimmed using Trimmomatic version 0.36 (49). The parameters employed were as follows. We removed leading and trailing nucleotides with quality below 10 (“LEADING” and “TRAILING” parameters, respectively). We used 4-nucleotide sliding windows and cut when average quality per nucleotide in a window was below 15 (“SLIDINGWINDOW” parameter), and we dropped any reads that were less than 40 nucleotides after this trimming (“MINLEN” parameter). Then we mapped the trimmed reads with the bwa-mem tool from BWA version 0.7.12-r1039 (50). The reference genome against which the reads were mapped was the CDC317 strain fasta file obtained in April of 2018 from the Candida Genome Database (51). We generated BAM files from this output using the SortSam and MarkDuplicates commands from Picard version 2.15.0 (http://broadinstitute.github.io/picard/). Finally, we called variants from these reads using Freebayes version 1.1.0-9-g09d4ecf (52) to jointly genotype all the strains involved. We filtered the SNPs using the vcffilter tool from vcflib (Garrison, https://github.com/vcflib/vcflib). We removed any SNPs for which the mean mapping quality (MQM) was below 30, the QUAL value was below 20 (indicating a probability of a false variant call greater than 0.01), and/or the read depth (DP) was below 30.

### Other computational methods.

Transmembrane helix predictions were calculated using the TMHMM (http://www.cbs.dtu.dk/services/TMHMM/) and PRO-TMHMM servers (http://topcons.cbr.su.se/) (53, 54). Sequences were aligned using the NCBI Protein BLAST online bioinformatic tool (https://blast.ncbi.nlm.nih.gov/Blast.cgi).

### Statistical analysis.

All statistical analyses were performed with GraphPad Prism v 6.0 software using parametric *t* tests or nonparametric Mann-Whitney tests. The values for the groups examined were considered statistically significantly different at *P < *0.05.

### Accession number(s).

Raw sequencing data are available under the BioProject ID PRJNA493002. Accession numbers for strains are as follows: wild-type CLIB 214, BioSample accession no. SAMN10120406; CAS^EVO^, SAMN10120407; AND^EVO^, SAMN10120408; MICA^EVO^, SAMN10120409.
