# Supplementary figures and images for: Echinocandin-Induced Microevolution of Candida parapsilosis Influences Virulence and Abiotic Stress Tolerance
Source: mSphere. 2018 Nov 14;3(6):e00547-18. doi: 10.1128/mSphere.00547-18 (PMC6236803; doi:10.1128/mSphere.00547-18)

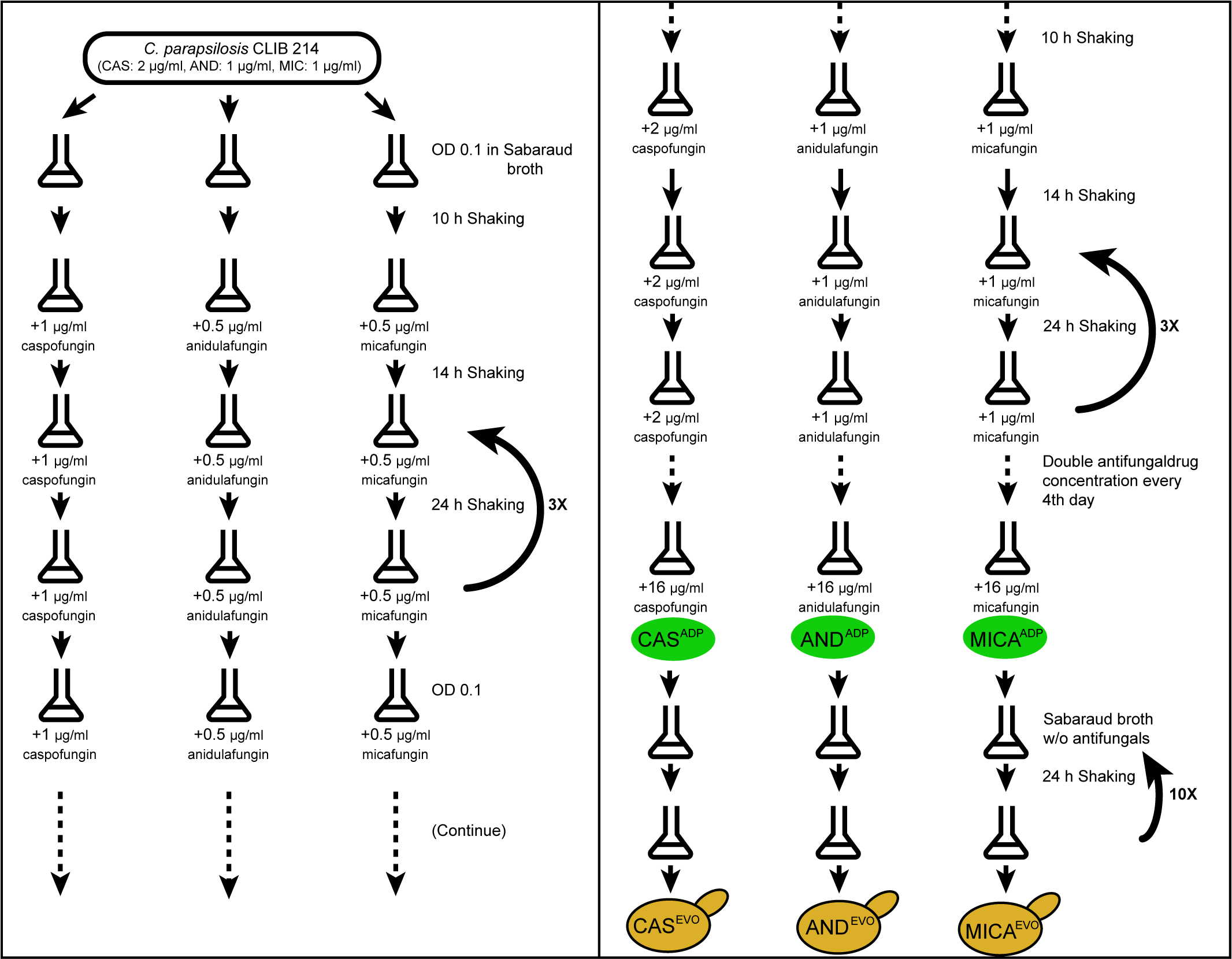

Supplement: FIG S1 [file sph006182702sf1.tif]
